# Room temperature electrically tunable rectification magnetoresistance in Ge-based Schottky devices

**DOI:** 10.1038/srep37748

**Published:** 2016-11-23

**Authors:** Qi-kun Huang, Yi Yan, Kun Zhang, Huan-huan Li, Shishou Kang, Yu-feng Tian

**Affiliations:** 1 School of Physics, State Key Laboratory of Crystal Materials, Shandong University, Jinan 250100, China; 2 Electrical & Computer Engineering, Harold Frank Hall, Room 4155, University of California, Santa Barbara, CA 93106-9560, USA

## Abstract

Electrical control of magnetotransport properties is crucial for device applications in the field of spintronics. In this work, as an extension of our previous observation of rectification magnetoresistance, an innovative technique for electrical control of rectification magnetoresistance has been developed by applying direct current and alternating current simultaneously to the Ge-based Schottky devices, where the rectification magnetoresistance could be remarkably tuned in a wide range. Moreover, the interface and bulk contribution to the magnetotransport properties has been effectively separated based on the rectification magnetoresistance effect. The state-of-the-art electrical manipulation technique could be adapt to other similar heterojunctions, where fascinating rectification magnetoresistance is worthy of expectation.

Silicon-based complementary metal oxide semiconductor transistors have achieved great success in modern semiconductor industry. However, the traditional development pathway is approaching its fundamental limitation, such as low electron mobility of Silicon, high leakage currents as the devices are becoming smaller, and large power consumption because of the high integration density. There is a growing interest in the development of spintronics devices with lower energy consumption^1^,^2^, where changing the resistance of a material and/or an artificial structure based on the manipulation of the spins of electrons by external magnetic and electric fields is crucial.

Modulation of resistance by an external magnetic field, i.e. magnetoresistance (MR) effect, has been a long-lived theme of research. Start from the first observation of anisotropic MR in 1857^3^, through the fast development of giant MR^4^,^5^, tunneling MR^6^ and colossal MR^7^ in the 20^th^ century, to more recent observation of emergent MR phenomenon, i.e., anomalous MR in nonmagnetic materials^8^,^9^,^10^,^11^,^12^, large MR in graphene devices^13^,^14^,^15^, spin Hall MR^16^,^17^, and rectification MR^18^, the study of MR has been full of surprise and several types of MR have already been used in commercial data storage technology. In particular, the recent observation of rectification MR in Al/Ge Schottky heterojunctions^18^ provides an alternative method for manipulating MR by using alternating current (AC).

Previously, electrical modulation of magnetotransport properties is usually realized through the usage of combined ferromagnetic, ferroelectric or multiferroic materials in both inorganic and organic heterostructures^19^,^20^,^21^,^22^, where electrical control of resistance, MR and exchange bias have been demonstrated. Another approach commonly used is based on spin transfer torque exerted by a spin polarized current on the spin moment of a nanometer scale magnet^23^,^24^. However, these experiments requires complex and high accuracy sample preparation techniques, such as nanometer size lithography, and the usage of magnetic materials have some disadvantages, such as the significant hysteresis, which sometime limit their practical applications. Moreover, the complex role played by the interface and bulk components in the magneto-transport properties are difficult to be separated since intricate coupling between spin, charge, orbital and lattice degree of freedom usually exists. In this means, effective and efficient ways toward electrical control of magnetoresistance are highly desired. Inspired by our recent observation of rectification MR under a pure alternating current^18^, we expected that simultaneously applying DC and AC to the rectification MR devices may hold promising potential to approach effective electrical control of magneto-transport properties. Because the rectification MR is a pure interfacial effect in nature, *i.e.*, the simultaneous implementation of the rectification and magnetoresistance of the Schottky interface under an applied AC current, it means that adding any pure resistance component with/without MR in series with the rectification MR devices could not affect the detected rectifying voltage while greatly influence the total resistance. As a result, simultaneously applying DC and AC during rectification MR measurements, the contribution from pure-resistance-like bulk component could be added to the detected DC rectifying voltage, hence a remarkable modification of the final rectification MR is expected. In the meanwhile, interfacial sensitive rectification MR measurements provide us a powerful tool to separate the interfaces contribution to the electrical transports from that of bulk components.

In this work, as a further extension of our previous rectification MR study, it is demonstrated for the first time that simultaneous application of DC and AC to the Ge-based Schottky devices is an effective way toward electrical control of rectification MR, where the rectification MR is tuned from −530% up to 32500%. Moreover, the interface and bulk contributions to the magnetoresistance are separated. The unique electrical manipulation technique could be adapted to other similar heterojunctions, which could accelerate the development of multifunctional spintronics.

## Results

### Electrical control of rectification magnetoresistance

**Figure 1:**
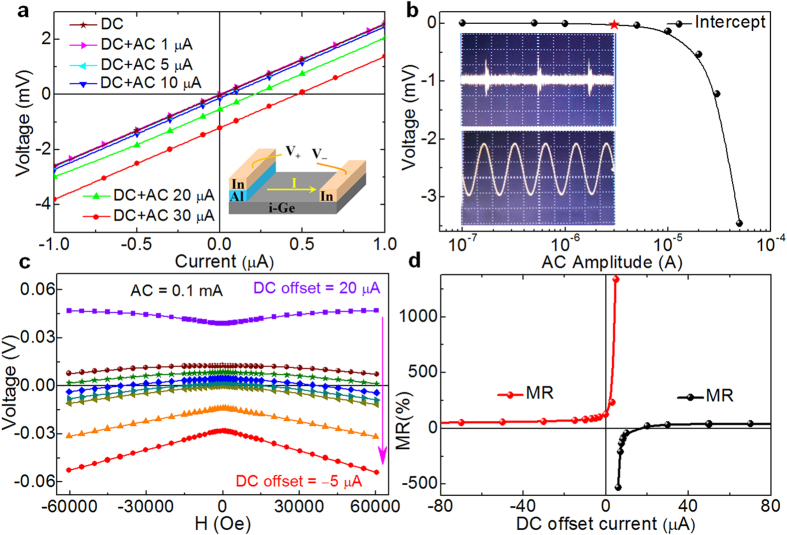
Electrical control of transport properties. **(a)** The conventional I-V curve measured by using Keithley 2400 and 2182A is marked as DC, while the I-V curves measured under different fixed AC amplitudes is marked as DC+AC x μA, where x represents the applied AC amplitude. Inset shows the schematic measurement configuration, where magnetic field is applied parallel to the current flow in the Ge substrate. **(b)** The AC amplitude dependence of the voltage intercept deduced from Fig. 1a. The top inset shows the real time voltage obtained by using oscilloscope when a small DC current of 1 nA was supplied by Keithley 2400, while the bottom inset shows the real time voltage obtained when a sinusoidal AC of 0.1 mA with constant DC offset of 1 μA was supplied by Keithley 6221. **(c)** The magnetic field dependence of the detected DC voltage measured for variable DC offset at a fixed AC = 0.1 mA. **(d)** The DC offset dependence of the deduced rectification MR from Fig. 1c.

Figure 1 summarizes the most intriguing results of current investigation, *i.e.*, great manipulation on the magneto-transport properties by the simultaneous application of DC and AC. First of all, a significant modification on the I-V curves by the application of AC is clearly revealed in Fig. 1a and b, where the voltage intercept (I_DC_ = 0) increases with increasing AC amplitude. This remarkable modification can be understood as a consequence of the conventional AC rectification effect by the Al/Ge Schottky device.

It is worthwhile mentioning that the voltage intercept of the Al/Ge Schottky device measured by using commercial Keithley 2400 and Keithley 2182A is about 22 μV, which is marked by a red ★ in Fig. 1b and significantly deviates from the ideal zero voltage. This phenomenon commonly occurs during small signal measurements if there exists any AC rectification, but it is usually neglected. This reminds us that we should be very careful when performing experiments at small current range, and this rectification voltage may greatly influence the spin motive force measurements^25^,^26^ and tunneling MR measurements^27^.

To understand the nonzero voltage intercept of the Al/Ge Schottky device measured by using commercial Keithley 2400 and Keithley 2182A, we further showed the real time voltage detected by using oscilloscope while a small constant DC current is supplied by Keithley 2400 (top inset of Fig. 1b). As comparison, an AC current with constant DC offset is supplied by Keithley 6221 (bottom inset of Fig. 1b). Though the shape of detected voltage is different for the two cases, a clear periodical voltage has been detected when the supplied DC is less than 10 nA as shown in top inset of Fig. 1b, which comes from the feedback signal of the equipment. This intrinsic feedback signal becomes undetectable when the current is above 100 nA. Therefore, it is confirmed that the feedback signal can produce a significant voltage intercept of the I-V curve during small signal measurements if there exists any AC rectification.

Figure 1c and d present the great tunability of rectification MR by the simultaneous application of DC and AC. Here, MR is defined as 
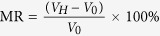
, where *V*_*H*_ (*V*_0_) is the DC voltage detected with (without) applied magnetic field. Figure 1c shows the influence of DC offset on the magnetic transport properties while keeping the AC amplitude fixed. Under the application of a pure alternating current, the rectifying voltage represents the average outcome of the corresponding real time voltage V_*H*_(*t*) over the time period *T* of the applied alternating current 
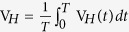
. Hence, depending on the device rectification direction, the detected rectifying voltage could be positive or negative though the real time voltage and current always have the same sign. Meanwhile, under the application of a pure direct current, the detected conventional voltage drop always follow the current flow direction. As a result, the total voltage detected during the rectification MR measurements under simultaneous application of DC and AC moves upward and even changes sign from negative to positive by applying a positive offset current, while it moves downward by applying a negative offset current. By controlling the applied DC and AC, the *V*_0_ could be tuned to near zero, which leads to the great enhancement of the detected magnetoresistance. Once the *V*_0_ changes from negative to positive around I_DC_  = 5.30 μA, the sign of the defined MR also changes. As summarized in Fig. 1d, not only extremely large rectification MR could be obtained, but also the sign of the rectification MR could be reversed by changing the DC offset. The rectification MR is 32500% when AC = 0.1 mA and DC offset current = 5.228 μA, while it is −530% when AC = 0.1 mA and DC offset current = 6.0 μA. This great tunability unambiguously indicates that simultaneous application of DC and AC is an effective method to manipulate the rectification MR of Schottky devices.

### Separation of interface and bulk transport properties

**Figure 2:**
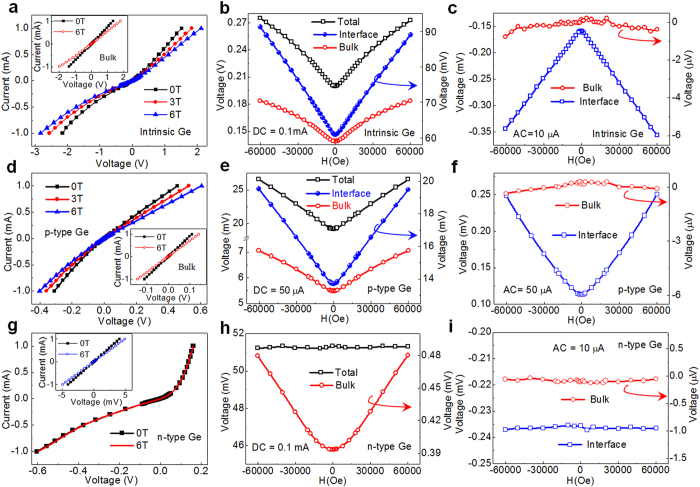
Transport measurements of devices grown on different substrates. **(a)–(c)** respectively show the corresponding I-V curves in different magnetic field (**a**), conventional DC MR (**b**), and AC rectification MR (**c**) for the intrinsic Ge-Schottky devices and Ge substrates. In (**b**) and (**c**), the bulk properties (marked by bulk) of the intrinsic Ge substrate have been measured after changing the Schottky electrode into Ohmic contact, where linear I-V curves have been confirmed as shown in the inset of (**a**). In (**b**), the interface DC voltage is obtained by subtracting the bulk signal from the total signal. In (**c**), the interface DC voltage of Ge-Schottky devices is directly measured under AC current. **(d)–(f)** are the same measurements as (**a**)–(**c**), but for the p-type Ge-Schottky devices and Ge substrates. **(g)–(i)** are also the same measurements as (**a**)–(c), but for the n-type Ge-Schottky devices and Ge substrates.

In order to go one step further towards a practical application of the rectification MR effect, the contributions from bulk layer as well as the interface to the magnetotransport properties have been separated by utilizing the interfacial nature of the rectification MR. Three types of Ge substrates with different resistivity has been used. The asymmetric I-V curves (Fig. 2a,d and g) were observed in all the as-prepared devices, indicating the existence of Schottky interfaces. As clearly shown in Fig. 2c,f and i, no rectification MR is observed after destroying the Schottky contact, meaning that bulk Ge substrate could not contribute to the rectification MR regardless its large difference in the resistivity. Rectification MR is only observed in p-type Ge-Schottky and intrinsic Ge-Schottky devices with much higher resistivity, suggesting that low carrier concentration is beneficial for the observation of rectification MR. This is consistent with previous report that shrinkage of carrier wave function under magnetic field is the physical origin of rectification MR^18^,^28^. Returning to the conventional DC magnetoresistance effect, it is reasonable to believe that the conventional DC voltages measured at the as-prepared Ge-Schottky devices (marked as total) contains the contribution from both the Schottky interface and the bulk Ge. After changing the Schottky contact into Ohmic contact, only the bulk signals could be measured as shown in Fig. 2b,e and h (marked as bulk). As a result, the interfacial contributions (marked as interface) to the conventional MR could be deduced by deducting the bulk signals from the total signals, assuming that bulk and interfacial resistance formed an equivalent series circuit. A linear magnetic field dependence of the interfacial conventional MR is obtained, suggesting that same physics lies behind the MR of both the interface and bulk components. In such a way, the interface and bulk contributions to the magnetotransport could be effectively separated by combing measurements under DC and AC. It should be pointed out that the positive conventional MR observed in n-type Ge (Fig. 2h) has a negligible influence on the interfacial MR (both total MR and interface MR is nearly zero), because its resistance is too small as compared with that of the Schottky interface.

### Anisotropic transport properties

**Figure 3:**
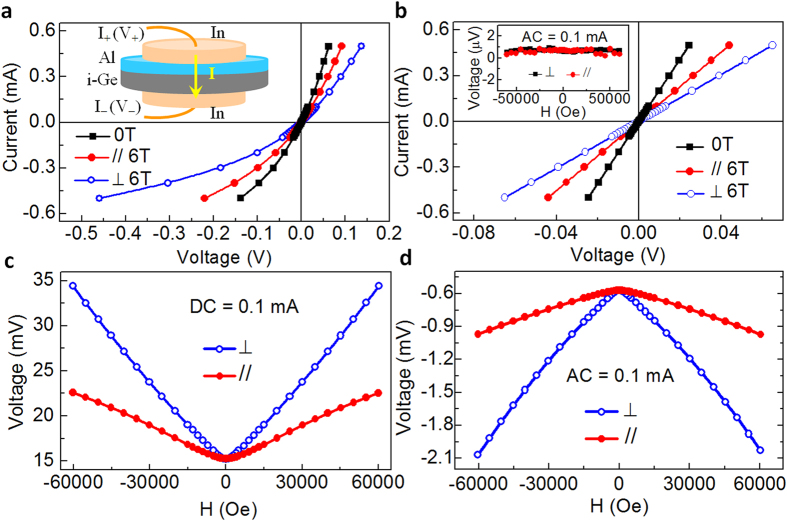
Anisotropic transport characterization. **(a)** The room temperature I-V curves of the studied circle Al/Ge Schottky heterojunction with the top electrode to be Schottky contacts. Inset reveals the schematic device configuration. **(b)** The room temperature I-V curves of the intrinsic Ge substrate with Ohmic contacts. Inset shows the corresponding rectification MR measurements. **(c)** and **(d)** respectively show the conventional MR and rectification MR measurements of the circle Al/Ge Schottky devices. Here, the ⊥ (//) configuration means that the applied magnetic field is perpendicular (parallel) to the current flow.

It is noticed that in the above studied stripe samples, the external magnetic field is applied parallel to the current flow in the bulk Ge substrate. It means that magnetic field is perpendicular to the actual current flow within the Schottky contact regime, where rectification MR comes from. Hence, these stripe structure can not clarify the dependence of the rectification MR on the relative direction between external magnetic field and the current flow. To achieve this goal, the Al/Ge Schottky heterojunctions as shown in the inset in Fig. 3a is used to ensure the current flows through the whole junction in a straight way. Figure 3a shows the room temperature I-V curves of the studied circle Al/Ge Schottky heterojunction. The enhancement of detected voltage under applied magnetic field indicates the positive magnetoresistance, and the significant asymmetry of I-V curves for the positive and negative voltage branch reveals the rectification effect. Here the most important feature is the significant anisotropy of MR, i.e., the I-V curves at 6 Tesla are different from each other for the perpendicular and parallel magnetic field configurations.

As a comparison, the property of the reference Ge substrate has been characterized after replacing the top Al/Ge Schottky contact by an Ohmic contact, where a symmetric linear I-V curves has been observed. As shown in Fig. 3b, the anisotropy of the conventional MR survives without Al/Ge Schottky junction rectification, demonstrating that the bulk Ge substrate itself also shows anisotropy in conventional MR. On the contrary, rectification MR completely disappears without Schottky junction rectification (the inset of Fig. 3b). This unambiguously indicates that rectification MR only originates from the Al/Ge Schottky junction, and both rectification and magnetoresistance are indispensable for the observation of rectification MR. Figure 3c and d further show the anisotropic behavior of the conventional MR and the rectification MR. It is clear that positive MR is observed for both conventional and rectification MR in either perpendicular or parallel configuration. Moreover, the perpendicular MR is always larger than the parallel MR within the studied current regime, highlighting the critical role played by the relatively configuration between magnetic field and electrical current.

The anisotropic behavior of conventional magnetoresistance could be understood as the traditional effect of the Lorentz force on the carrier motion. When the applied magnetic field is perpendicular to the current, the curving of the carrier trajectory is much larger than in the parallel case, leading to stronger perpendicular magnetoresistance^28^. The above mechanism of the anisotropic transport is also applicable to the rectification MR, since rectification MR and conventional MR share similar magnetic field dependence^18^ and the voltage detected in rectification MR represents the average outcome of the corresponding real time voltage over the time period of the applied alternating current. So it is easy to understand that the rectification MR (*i.e.*, the average outcome of real time voltage) is anisotropic if the conventional MR (*i.e.*, the real time voltage) is anisotropic.

## Discussion

Finally, it should be pointed out that the voltage detected during rectification MR measurements under pure AC could be either positive (Fig. 2f) or negative (Figs 2c and 3d), which is determined by the device-dependent rectification direction. On the contrary, the voltage detected is always positive along the current direction in the conventional MR measurements under pure DC. Therefore, the total voltage detected during rectification MR measurements could be moved upwards or downwards when the DC and AC are applied simultaneously, resulting the observed significant tunability of rectification MR. Another important thing need to be kept in mind is that rectification MR requires the simultaneous implementation of both rectification and magnetoresistance in the same devices. Even more exciting is that this distinctive electrical control technique could be operated in either non-magnetic or magnetic systems with remarkable rectification MR, such as magnetic tunnel junctions with asymmetrical tunneling barriers, Schottky or magnetic diodes with remarkable magnetoresistance effect, which may open an alternative way towards multifunctional spintronics.

To summarize, the present experiments illustrate that applying direct current and alternating current simultaneously to the Al/Ge Schottky devices is an effective and efficient way to realize electrical control of rectification magnetoresistance, where both the sign and amplitude of the rectification MR could be significantly controlled. Moreover, the interface and bulk contribution to the magnetoresistance could be well separated in these Schottky devices, and the anisotropic transport behavior provides us an additional operation degree of freedom to manipulate the rectification MR. Most importantly, the state-of-the-art electrical manipulation technique could be applied to other similar heterojunctions, where fascinating rectification magnetoresistance is worthy of expectation.

## Methods

### Sample preparation

During the experiments, different types of single side polished Ge substrates are used, *i.e.*, <100> orientated intrinsic Ge with resistivity of 55.6~59.4 Ωcm is marked as intrinsic Ge; <100> orientated p-type doped Ge with resistivity of 3.2~3.5 Ωcm is marked as p-type Ge; and <111> orientated n-type doped Ge with resistivity of 0.02~0.15 Ωcm is marked as n-type Ge. Unless specifically noted otherwise intrinsic Ge substrate is used. For the electrical manipulation studies, the sample is designed to be a strip structure (5 mm in length and 2 mm in width) as shown in the inset of Fig. 1a. For the anisotropic transport studies, the sample is designed to be a circle structure (diameter equals to 3 mm) with two electrodes attached on the top and bottom of the structure as schematically shown in the inset of Fig. 3. In both cases, the In/Al/Ge electrode is designed to be Schottky contact, where the Al layer with a thickness of 100 nm is prepared by magnetron sputtering at room temperature and In contact is prepared by using solider iron. And the In/Ge electrode is designed to be Ohmic contact. In order to form a more ideal connection, Gallium Indium Tin eutectic (Ga:In:Sn = 62:22:16 wt%) is used to react with the substrate for 30 minutes before the In electrode is prepared by using solider iron.

### Electrical characterizations

The conventional MR and I-V measurements were performed by using two points method with Keithley 2400 electrical current source meter and Keithley 2182A voltage meter. The current flows from Schottky electrode to the Ohmic electrode is always defined as the positive current direction. The I-V curves under fixed AC amplitudes were measured by using Keithley 6221 source meter to provide a sinusoidal AC input with different DC offset and Keithley 2182A voltage meter to detect the generated DC voltage. The rectification MR with or without a constant current offset was also measured by using two points method with Keithley 6221 source meter and Keithley 2182A voltage meter. The frequency of the applied AC is fixed at 1 kHz for all the measurements. During the MR measurements, the applied magnetic field is parallel or perpendicular to the current flow in the anisotropic study while it was applied in the film plane and parallel to the current direction of the Ge substrate in the other experiments.

## Additional Information

**How to cite this article**: Huang, Q.-K. *et al*. Room temperature electrically tunable rectification magnetoresistance in Ge-based Schottky devices. *Sci. Rep.* **6**, 37748; doi: 10.1038/srep37748 (2016).

**Publisher's note:** Springer Nature remains neutral with regard to jurisdictional claims in published maps and institutional affiliations.
